# *Salmonella* in the food chain within the Gulf Cooperation Council countries

**DOI:** 10.3934/microbiol.2024023

**Published:** 2024-06-25

**Authors:** Mohamed-Yousif Ibrahim Mohamed, Ihab Habib, Hazim O. Khalifa

**Affiliations:** 1 Department of Veterinary Medicine, College of Agriculture and Veterinary Medicine, United Arab of Emirates University, Al Ain P.O. Box 1555, United Arab Emirates; 2 ASPIRE Research Institute for Food Security in the Drylands (ARIFSID), United Arab Emirates University, Al Ain, United Arab Emirates

**Keywords:** *Salmonella*, food chain, foodborne infection, Gulf Cooperation Council countries, one health

## Abstract

Infections caused by bacteria originating from tainted food sources are a widespread concern due to their large economic impact and detrimental effects on public health. We aimed to explore literature focusing on the presence of *Salmonella* in the food supply chains of Gulf Cooperation Council (GCC) countries and to provide an overview of available information concerning health-related issues and the status of salmonellosis in humans in GCC countries. The reviewed evidence underscored a gap in our comprehensive understanding of the prevalence of *Salmonella* in the food supply of GCC countries. Molecular characterization efforts to pinpoint the sources of *Salmonella* in these nations were limited. Surveys targeting *Salmonella* in the food supply of GCC countries have been infrequent. While qualitative data indicated the presence or absence of *Salmonella*, there was a noticeable lack of quantitative data detailing the actual quantities of these bacteria in chicken meat supplies across GCC countries. Although reports regarding *Salmonella* in animal-derived foods were common, the literature highlighted in this review emphasized the persistent challenge that *Salmonella* pose to food safety and public health in GCC countries. Addressing this issue requires concerted efforts to enhance surveillance, improve control measures, and promote greater awareness among stakeholders in the food supply chain.

## 1. Introduction

Internationally, recurrent occurrences of foodborne pathogens are a challenge to food safety and trade. Foodborne illnesses constitute a major global health concern that is evident in Europe and many other countries [1]. As per reports from the European Centre for Disease Prevention and Control (ECDC) and the Zoonoses Reports by the European Food Safety Authority (EFSA), there are approximately 93.8 million instances of human enteric illnesses due to *Salmonella* infection annually, leading to an estimated 150,000 deaths [2]. Salmonellosis has been ranked as the leading cause of foodborne illnesses in the United States, the Foodborne Diseases Active Surveillance Network (FoodNet) of the Centers for Disease Control and Prevention (CDC) has reported an uptick in hospitalizations due to salmonellosis and campylobacteriosis in recent years [3].

Based on a World Health Organization (WHO) report, the estimated burden of foodborne diseases per population in the Middle East and North Africa region stands to be third highest globally, only surpassed by the African and South-East Asian regions [4]. The study also declared that about 70% of the total foodborne diseases in this area are caused by *Campylobacter*, *E. coli*, non-typhoidal *Salmonella* (NTS), and Norovirus [5],[6], enforcing the fact that these agents are a significant source of foodborne illness in this region. Nevertheless, the true prevalence of foodborne infections in the Middle East is rather a difficult task to determine due to the lack of cohesive surveillance systems. These efforts are important for monitoring occasional cases, controlling outbreaks, and supplying isolates that are essential in source identification and risk analysis on the national and regional level [7]. Besides demanding a more accurate data collection regarding the occurrence and infection by pathogens, there is also an incomplete picture of antimicrobial resistance among bacterial agents associated with foodborne infections across the Middle East region. However, despite some research having shown different patterns in the relationship between man and food [7],[8], a concrete understanding of this problem has not been elaborated.

The GCC countries have such organizations as the Ministry of Health or any other organizations focusing on implementing public health programs [9],[10]. In Bahrain, acting as a health-protective institution, the role of the Ministry of Health is to prevent and control diseases, promote and educate the healthy lifestyle, facilitate and conduct public health research as well as to gather statistical information, regulate the healthcare system, and to respond to the sharp threats to public health [11]. Kuwait's Ministry of Health (MOH) is responsible for disease prevention and control and health promotion, education and communication, vaccination, environmental health, and emergency and disaster Programs [12]. In Oman, the Ministry of Health is charged with the responsibility to formulate policies and strategies on public health, conduct epidemiological surveys and investigations, implement intervention measures, promote the issues of health education and environmental health, and coordinate issues relating to emergency preparedness and response [13]. In Qatar, the Ministry of Public Health (MoPH) technical guidance and support on health promotion and disease prevention, infection and chronic disease monitoring, disaster and outbreak management, as well as health service standard control [14]. The Saudi Center for Disease Prevention and Control (Saudi CDC) in Saudi Arabia a government organization for the prevention and control of disease across the region, research in the field of public health, promotion of health with regards to disease control and management, formulation of policies and guidelines, and disaster management [15]. In the United Arab Emirates, the Ministry of Health and Prevention (MOHAP) is tasked with the responsibilities of developing health policies, regulation and monitoring of diseases and promotion of health and prevention of diseases, education and campaigns, and regulation and supervision of health facilities [5].

Some of the core functions of public health agencies in GCC countries include disease identification, tracking and reporting of Infectious diseases, and use of strategies to prevent or contain spread of such diseases. They also impart health education with awareness of different health issues as well as encourage the people to embrace healthy lifestyles [6]. Policy and regulation are important as these agencies formulate health policies and standards and regulate healthcare providers and services [10]. Another broad category is research and data gathering, which entails engaging in public health research to influence policy and practice and also gathering health data for decision making [16]. Another significant area of focus is emergency preparedness and response, as these agencies develop plans and provide services in preparation for and in response to public health emergencies, including diseases and calamities. Furthermore, immunization activities are carried out to prevent the occurrence of vaccine-preventable diseases within the population. These agencies work in unison within their countries and across the whole GCC in a bid to enhance the quality of life of their people [10].

In GCC countries most of the food is retailed in the formal chain which includes supermarkets, hypermarkets, grocery shops, retail outlets among others. This sector is relatively developed, many big stores and international processors lawfully maintain high-standard food hygiene and quality requirements which are supervised by the government [17]. However, there are street vendors, small and irregular markets, and local food stalls, which are informal food sectors and occupy significantly fewer food sales than the formal sector. This sector is relatively unstandardized and does not always follow the high standards of food hygiene and quality that are expected [10]. Currently, large formal retail holds the largest share in the UAE, Saudi Arabia, and Qatar due to higher urbanization, economic development, and preference for security. These countries have developed modern retail facilities and they prefer supermarket and hypermarkets [18]. For instance, in the UAE, the modern retail formats already cut across food retail where many multinational supermarket chains are found. Overall, it is widely believed that the formal sector's share ranges from 80–90% of overall food sales in most GCC countries, while the informal sector makes up the remaining 10–20% [19]. There is a clear tendency to institutionalize the sector, which can be observed due to economic development, urbanization, new legislative requirements ensuring the quality and safety of food products for the population's health and economic renewal.

The contamination of *Salmonella* in the food chain is a public health concern in Iraq, Syria, Jordan, Lebanon, Iran, and North African states [5]. In Iraq and Syria, the instability and conflict have severely impacted food safety infrastructure. The breakdown of regulatory systems and limited access to clean water and sanitation have exacerbated the risk of *Salmonella* contamination [9]. The likelihood of *Salmonella* infection is increased by inadequate handling of foodstuffs and lack of cold chains in storage. Street food and locally produced dairy and meat products are particularly susceptible to contamination [20]. Jordan is steadily increasing its pace to comprehensively address the food safety issue, but certain hurdles exist. One of them is that the country relies on the importation of food, thus it is important to check on the quality of foods that are imported [6]. *Salmonella* has been linked to the consumption of imported poultry eggs and other locally produced foods. The Jordanian government has adopted stringent food safety measures, though the application is usually not very thorough, especially in the countryside [21]. Similar challenges are experienced in Lebanon, especially in food safety, and compounded by economic challenges and a less enhanced regulation system [22]. *Salmonella* has been identified in the flow chain and associated with imported and locally produced foods [5]. The challenge of hygiene is a concern, especially for the informal sector food vendors such as street vendors and mini food producers. Continued measures have been taken towards improving food safety but some barriers need to be addressed in ensuring full and proper compliance [22]. Iran has invested more in food safety than some developing countries but issues with *Salmonella* persist [23]. The country has experienced many episodes arising from contaminated chickens, eggs, and milk [24],[25]. Peculiarly, there are very strong food safety laws, however, implementation varies across regions. Rural areas often face greater challenges due to less stringent enforcement and limited access to modern food safety practices [26]. In North African countries, such as Egypt, Libya, Tunisia, Algeria, and Morocco, *Salmonella* contamination is a concern [5]. They have a composite structure of food production methods with both modern and traditional techniques [9]. Street food is widely consumed, and contaminated food items due to improper hygiene practices are common. Poultry and egg products are a main reservoir of *Salmonella* [27]. Some measures have been taken towards enhancing the standards of food viability but due to some constraints such as the economic and infrastructural developments the process is slowed [28].

This review presents updated data on the epidemiology of *Salmonella*, a common cause of foodborne infections worldwide among humans. The methodology involved a systematic literature review to gather, evaluate, and analyze relevant literature. Individual studies were analyzed independently to identify patterns and trends related to the research question. Author extracted characteristics such as publication year, research methods, and outcomes for quantitative analysis [29]. Searches were conducted on platforms like PubMed, Science Direct, Scopus, Web of Science, and Google Scholar, along with exploration of postgraduate theses and national reports. The focus was on foodborne infections in diverse food types across GCC countries over the past two decades. The GCC region includes Bahrain, Kuwait, Oman, Qatar, Saudi Arabia, and the United Arab Emirates (UAE), with a combined coastline along the Arabian Gulf spanning approximately 2,200 kilometers (1,367 miles).

*Salmonella*, slender Gram-negative bacteria within the Enterobacteriaceae family, represent a frequent zoonotic pathogen accountable for causing foodborne illnesses globally. Human infection with *Salmonella* often occurs through the consumption of contaminated animal-derived foods like beef, poultry, pork, eggs, and milk. Contact with animals such as reptiles and turtles are also linked to NTS transmission [1],[30]. Surprisingly, outbreaks of salmonellosis in humans have been associated with fruits and vegetables contaminated with animal feces [31]. The authorities in Bahrain, Kuwait, Oman, Qatar, and the United Arab Emirates have collectively agreed to implement a uniform guideline regulating the criteria for Halal animal slaughter in accordance with Islamic Law (Shari'a), as specified by their individual national standards [32].

We seek to clarify recent advancements derived from published research over the last twenty years concerning the presence of *Salmonella* within the food chain and its pathogenicity in the GCC nations.

## 2. Current status of human salmonellosis in GCC countries

Salmonellosis is a major public health issue that is experienced in the global communities including the GCC countries. The incidence and prevalence of salmonellosis differ in these countries by seasons, climate, food security, and health standards [33]. Salmonellosis is not prevalent as an endemic disease in the GCC countries but is experienced as isolated cases or occasional epidemics due to contaminated poultry, eggs, dairy, and produce. Since the GCC countries' population consumes a significant portion of imported food, it is important to regulate food imports to reduce the likelihood of *Salmonella* [9],[34].

The governments and health authorities of the GCC member countries have taken steps to curb and treat *Salmonella* infections. This can involve regulation of food safety, implementing programs that provide checks on the cases of foodborne illnesses, consumer education, and applying laws that deal with hygiene in food production areas [10]. Healthcare systems in GCC countries are equipped to diagnose and treat *Salmonella* infections. Early treatment including fluid replacement therapy and in cases of severe diarrhea, antibiotics also help in cutting down the length and intensity of the disease. Given the interconnected nature of global trade and travel, they also address this public health problem through regional and international cooperation. Exchange of information, and collaboration in research activities, and alignment of food safety measures can improve the prevention and control measures.

Nonetheless, *Salmonella* infection control remains a challenge due to inadequate food safety legislation, restricted monitoring, and antibiotic-resistant strains. Public awareness, food safety measures, or recurrent attention to continued rising incidences of salmonellosis, especially in the GCC countries [35]. New knowledge in preventive measures and treatments can be useful in the process of enhancing the general condition. More strategies are needed to contain the spreading of *Salmonella* and enhance the health status of the population. It can therefore be realized that this is a global health issue that requires concerted efforts of governments, healthcare professionals, and partners from the international community [10].

## 3. Sources of *Salmonella* in GCC countries

*Salmonella* is widely recognized as one of the most prevalent zoonotic pathogens associated with foodborne illnesses worldwide. Most human infections result from the consumption of food of animal origin, including beef, poultry, pork, eggs, and milk, which get contaminated. Additionally, direct contact with animals such as reptiles and turtles can contribute to the transmission of *Salmonella* [1],[30]. Infections from human-salmonellosis have also been associated with fruits and vegetables that get contaminated with animal feces [31],[36].

The identification of the presence of *Salmonella* in the foods is essential to understanding the risk of foodborne illnesses in the GCC countries. According to a recent meta-analysis by Al-Rifai et al. [7], out of 252,831 individuals tested, 6,356 showed NTS positivity. The pooled *Salmonella* prevalence in the Middle East and North Africa was estimated at 6.6% (95% confidence interval (CI): 5.4%–7.9%). According to their study, NTS prevalence in foods consumed in the Middle East and North Africa region has been established at a general average of 8.8%. Among animal-based foods, the pooled prevalence was 9.0%, while fishery products and plant-based foods had a pooled prevalence of 22.9% and 0.4%, respectively. The most common serotypes in the region were identified as S. Typhimurium (28.0%), S. Enteritidis (23.6%), and S. Kentucky (20.3%). However, despite these findings, there remains a lack of comprehensive understanding regarding the presence of NTS in the food supply chain of Arab countries, with limited efforts made towards molecular characterization to identify sources of this significant pathogen [10],[35].

**Figure 1.**
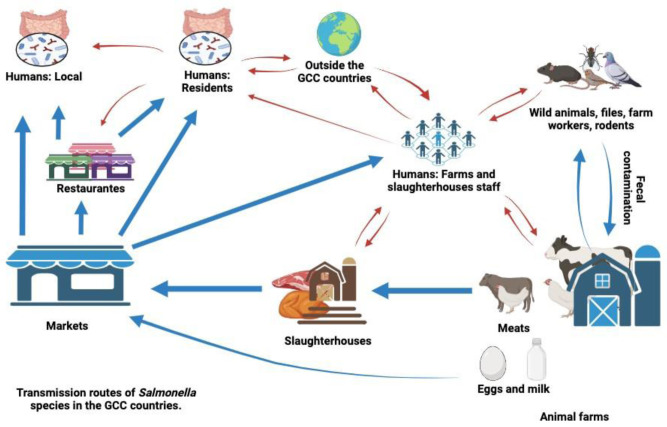
Displays both the direct and indirect routes of *Salmonella* transmission to humans in GCC countries. Red arrows: direct routes; Blue arrows: indirect routes. Created using Biorender.com (accessed on February 24, 2024).

*Salmonella* can invade the human body indirectly or directly. Indirect transmission of *Salmonella* occurs through various channels, such as contaminated food, water, and the environment. This bacterium can infect as diverse foods as poultry, eggs, meat, dairy products, fruits, and vegetables. Ingestion of the contaminated foods results in ailment. Direct transmission is more prevalent among people in the business of handling animals or animal-derived products [37],[38]. Some of the occupations include farming, veterinary, slaughtering, processing of poultry products, and abattoirs. Another avenue of transmission is through direct contact with the *Salmonella*-infected recombinant pets including puppies or kittens with symptoms of *Salmonella* diarrhea. There are many ways through which diseases can be transmitted from animals to humans but the common channels normally include eating of infected meat, eggs, or milk [1],[39]. As shown in Figure 1 below, the transmission of *Salmonella* to humans in the GCC countries may take several possible pathways.

*Salmonella* can be harboured and disseminated in foods through contaminated human food handlers who consequently transmit it to consumers. When handling food products that are contaminated with *Salmonella*, personnel who come into contact with this bacterium are at a higher risk of getting infected thus there is a risk of cross-contamination and exposing consumers to the bacteria [38]. Infection control measures are torn in such facilities and hence practicing good hygiene and sanitation can help to reduce the risk of passing the bacteria. Notably, low-grade exposure at different time intervals leads to partial tolerance gradually, which can help cut down on manifested symptoms after contracting the disease [40]. This immunity also influences food safety measures adopted by the food industry, to ensure that knowledgeable employees avoid health risks such as correct hygiene measures, food sanitation processes, and adherence to the food safety policies [41]. Comprehensive knowledge and control of occupational exposure to *Salmonella* will contribute to the development of healthy employees, as well as the improvement of the quality of food production and agriculture. Table 1 provides an overview of recent research on *Salmonella* prevalence in food in GCC countries.

**Table 1.** The presence of *Salmonella* in food across Gulf Cooperation Council countries.

Country	Tested food samples (Total number)	Size and type of sample analyzed	% of *Salmonella*-positive samples or isolates No.	Confirmation methods	*Salmonella* serotypes (%) *	References
Bahrain	Imported fish (*n* = 223)	25 g homogenate in 225 mL buffered peptone water (BPW)	89 (39.9%)	API 20E strips	ND	[34]
Kuwait	Chicken meats (*n* = 2064)	10 g homogenate in 90 mL BPW	16.2	API 20E strips	Typhimurium (29.6), Enteritidis (40.9), Thomson (22.1), Munchen (7.4)	[42]
	Chicken meats (*n* = 360)	10 g homogenate in 90 mL BPW	6.1	Biochemical tests	Enteritidis (100)	[43]
	Eggs (*n* = 30)		10		Enteritidis (100)	
Oman	Imported frozen chicken (*n* = ND)	10 g homogenate in 90 mL BPW	123 isolates	API 20E strips	ND	[44]
	Live chickens from private chicken farms (*n* = ND)		191 isolates		ND	
	live chickens from commercial chicken farms (*n* = ND)		201 isolates		ND	
Qatar	Chicken meats (*n* = 270)	Each chicken carcass was soaked in 250 mL of sterilized buffered peptone water	11.1	PCR technique	ND	[45]
	Beef (*n* = 40)	Enriching 10 g of the sample in 100 mL of Tetrathionate broth	20	PCR technique	ND	[14]
	Chicken (*n* = 21)		23.8		ND	
	Mutton (*n* = 30)		20		ND	
	Seafood (*n* = 62)		0		ND	
	Cheese (*n* = 11)		0		ND	
	Ready to eat foods (*n* = 55)		9.1		ND	
	Surface (*n* = 68)		22.1		ND	
Kingdom of Saudi Arabia	Shawarma (*n* = 100)	25 g homogenate in 225 mL BPW	20	PCR technique	Typhimurium (25), Enteritidis (75)	[46]
	Chicken meats (*n* = 50)	55 g in 225 mL Lactose broth	66	PCR technique	Typhimurium (43.5), Other serotypes (56.5)	[47]
	Shawarma (*n* = 80)	25 g homogenate in 225 mL BPW	10	API 20E strips	Typhimurium (62.5), Enteritidis (37.5)	[36]
	Falafel (*n* = 25)		12		Typhimurium (66.7), Enteritidis (33.4)	
	Vegetable salad (*n* = 35)		22.9		Typhimurium (75), Enteritidis (25)	
	Kibtha (*n* = 15)		26.7		Typhimurium (50), Enteritidis (50)	
United Arab Emirates	Fresh Salad Vegetable (*n* = 400)	25 g homogenate in 225 mL BPW	1.3	PCR technique	ND	[48]
	Chicken meats (n = 315)	25 g homogenate in 225 mL BPW	41.6	PCR technique	ND	[49]
	Chicken meat (*n* = 60)	5 g homogenate in 50 mL distilled water	46.7	VIT-*Salmonella* kit (Vermicon Identification Technology,Munic, Germany)	ND	[50]
	Tabbouleh, hummus, Greek salad, and coleslaw (*n* = 120)	25 g in 225 mL BPW	0	Biochemical tests	ND	[51]

* The percentage (%) of *Salmonella* serotypes is calculated from the positive samples (isolated target bacteria).

In Bahrain, potential risk factors affecting conformity with risk factor criteria for *Salmonella* infections are water quality or supply, low birthweight, nationality, gender, age, geographical location, and gastrointestinal diseases [52]. A cross-sectional study performed in 2012–2013 reported the presence of *Salmonella* in mackerel that was imported into Saudi Arabia [34]. Kuwaiti studies showed *S. enterica* entering the human food chain through slaughterhouses, with predominant serotypes S. Enteritidis and S. Typhimurium and varying antimicrobial susceptibilities [43]. Overall knowledge about food safety by Kuwaiti restaurant food handlers was quite reasonable, but there were some shortcomings in their knowledge, emphasizing the necessity of enhancing the level of training [43],[53]. Different types of *Salmonella* strains were isolated from various regions in Oman through research, from frozen chicken carcasses, to live chickens from farm and sewerage water [44]. The studies conducted in Qatar identified the presence of *Salmonella* in raw chicken carcasses and other retail products [14],[45]. Qatar's Supreme Council of Health and Ministry of Municipality and Urban Planning forward the initiative to promote cleanliness in restaurants and ensure high standards of food laws [14],[45]. Saudi Arabia has also established a national reporting policy on cases of bacterial foodborne illnesses with *Salmonella* outbreaks recurring across regions and food items [10],[35]. Studies conducted in Saudi Arabia detected *Salmonella* in ready-to-eat fast foods and chicken carcasses [36],[46],[47]. Recent research in the United Arab Emirates (UAE) uncovered *Salmonella* prevalence in chicken meat samples, in addition to contamination in salad vegetables, and water from parks used for irrigation [48]–[51],[54].

Evaluating national or regional conditions concerning *Salmonella* in the GCC requires a systematic evaluation of several factors influencing the prevalence, distribution, and management of *Salmonella* infections across member countries. This evaluation is best captured by aspects such as epidemiological surveillance, risk factors, food safety regulations, veterinary and agricultural practices, public health infrastructure, and international collaboration. Through such an assessment, GCC countries could accurately determine deficiencies, focus their efforts on most important areas, and enhance their united capacity to reduce the extent of public health harm that may result from *Salmonella* infections.

## 4. Human salmonellosis in GCC countries

*Salmonella enterica* remains an important cause of gastroenteritis in developed countries [2]. The prevalence of salmonellosis affects public health interest in numerous European countries [1]. Salmonellosis in human can manifest as mild to moderate, acute gastroenteritis often limited to self-treatment, and might be characterized by a wide range of symptoms including abdominal pain, diarrhea, vomiting and/or cramps, fever, and nausea. However, severe cases leading to fatalities can occur [55]. In some cases, *S. enterica* may spread beyond the gastrointestinal tract, causing localized or invasive infections, this occurs among immunocompromised persons, the elderly, or children. People working directly with animals or animal products, such as veterinarians and farmers, poultry processors, animal slaughterers, and butchers are at a higher risk due to direct exposure in their line of duty [5],[56]. *Salmonella* infections have an ability to provide immunity against the infections within human body and therefore less hospitalization and mortality rates observed. This could be due to immune reactions elicited by previous contact with *Salmonella* [57]. As a result, individuals with acquired resistance may develop less severe symptoms or no symptoms at all, thus minimizing their chance of hospitalization and fatalities. However, although resistance helps to reduce the impact that *Salmonella*-related diseases can have in some cases, it does not thoroughly eradicate the risks and may present certain difficulties when it comes to the administration and treatment of such diseases [58]. Emphasizing importance is necessary, considering the significant lack of research on salmonellosis in humans across GCC countries. This is why it is important not to leave this gap unaddressed any longer.

Table 2 outlines the outbreaks and studies conducted on *Salmonella* in GCC countries captured in this review, findings obtained, and measures adopted in controlling the challenges posed by these events to public health. In Bahrain, although more information regarding the outbreak has not been availed, the involvement of eggs and mayonnaise pinches the finger at *Salmonella* and specifically S. Enteritidis in line with, other comparable cases across the world [2],[5]. Data from The Kingdom of Bahrain Ministry of Health (KBMH) website having analyzed the frequency rate of salmonellosis from the year 2012 to 2020 indicates a cyclic occurrence with the incidence rate rising in the year 2014 and reaching 26. 675 cases and 24. 492 in 2015 [11]. The 2014 outbreak originated from a restaurant that was later cited for having *Salmonella* in food samples. Subsequently, the involved restaurant and two branches were shut down until they met the set health requirements [10]. An example of foodborne illness in Kuwait occurred in July 2018 when over 287 people were affected after taking a meal from a falafel restaurant in Hawally [59]. The medical reports showed that all the served food items were positive for *Salmonella*, especially mayonnaise which was exposed to poor hygiene and handling, as well as improper storage and handling conditions. Likewise, in July 2018, American soldiers at Camp Arifjan suffered from a *Salmonella* outbreak, which was traced from the food vendors. Although there were difficulties in isolating the source of the pathogen, a survey was conducted that required inspections of dining and medical facilities, and all employees were required to participate in a brief sanitation and food handling reminder [60]. This led to the detection of stools with bacterial enteropathogens among the American soldiers who sought medical attention of fifty percent [61]. Research conducted in Kuwait shows that *Salmonella* is common; blood cultures pointing to NTS infections are also seen [62]. In Oman, gastrointestinal illnesses often involve *Salmonella* infections. Outbreaks identified in northern Oman indexing to a restaurant showed isolations of *Salmonella* from the clients, food handlers, and samples calling for an urgent public health intervention [63]. In Ibri province, contaminated food educed diseases among consumers, hence observing the laws governing the protection of consumers [64]. Workers at Oman's Petroleum Development camp were affected by a food poisoning incident which led to collaboration between the company and health authorities to determine the cause [65]. According study conducted in Dhahira revealed microbial etiology of diarrhea in children with a special spotlight on *Salmonella*, *Shigella*, and *E. coli* (Table 2) [66].

Chang et al. [67] discussed in more detail the outbreaks of *Salmonella* in both hospitals in Qatar and food animals isolating 27 different sequence types as well as frequently circulating serovars such as S. Enteritidis and S. Typhimurium. In another study, Peters et al [68] related gastroenteritis in the health situation of Hamad Medical Corporation to high *Salmonella* rates. An outbreak that occurred in a hospital in Doha later identified *Salmonella* group D, pointing more to the concerns in healthcare facilities. A large outbreak was documented by Nazzal et al. [69] where Filipino Mess Hall was affected and mayonnaise salad was linked to the cases. Qatar's Ministry of Municipality and Urban Planning (MMUP) ramped up checks and some businesses faced fines for hygiene lapses in food outlets. In Saudi Arabia, Alsayeqh [15] used data gathered from the MoH departments and health centers to investigate demographic features and spatial distribution of salmonellosis. Somily et al. [70] investigated the diarrheal species in Riyadh with *Salmonella* spp. predominant among young patients. Another study was done by Elhadi et al [71] in 2014 in Dammam identifying the prevalence of NTS and Johargy et al. [72] in 2016 discovered a high percentage of *Salmonella* in children admitted to hospitals in Makkah and Jeddah. It would be useful to expand on the types of cross-contamination and contamination risks involved with cultural practices in wedding ceremonies, as mentioned by Aljoudi et al. [73] The inspections conducted in the UAE indicated several distinctions in the pathogens present including *Salmonella*.

**Table 2.** Summarizing the outbreaks and studies related to *Salmonella* in GCC Countries.

Country	Outbreak/Study and time	Hospitalization	Key findings	Implemented measures	References
Bahrain	2014 (from January to December)	26,675	Salmonellosis outbreak associated with a restaurant	Closure of implicated restaurant and associated branches	[11]
Kuwait	2018 (July)	287	Salmonellosis outbreak associated with a restaurant	ND	[59]
	2018 (July)	45	The outbreak was linked to food vendors	ND	[60]
	2007	432	The outbreak was linked to food vendors	The majority were hospitalized but later discharged	[61]
	2006 to 2020	700	Gastroenteritis diagnosed as salmonellosis	ND	[12]
	1988	98	Children gastroenteritis diagnosed as salmonellosis within a span of 15 months	ND	[62]
Oman	2015 (August 27 and September 2)	50	Salmonellosis outbreak associated with a restaurant	ND	[63]
	2000 to 2002	18	Children gastroenteritis diagnosed as salmonellosis	ND	[66]
Qatar	2011 (August) to 2014 (May)	128	Gastroenteritis diagnosed as salmonellosis	ND	[68]
	2010 (December 6)	51	Salmonellosis outbreak associated with a restaurant	ND	[74]
	2010 (November 17)	300	Salmonellosis outbreak associated with a restaurant	A fine imposed on the restaurant	[69]
Saudi Arabia	2005 (January) to 2010 (December)	491	Gastroenteritis diagnosed as salmonellosis between 2005 and 2010	ND	[70]
	2008 (September) to 2011 (April)	158	Gastroenteritis diagnosed as salmonellosis between 2008 and 2011	ND	[71]
	2010	9	Children gastroenteritis diagnosed as salmonellosis	ND	[72]
	2010	40	Salmonellosis outbreak associated with a restaurant	A fine imposed on the restaurant	[73]
UAE	2007 (April) to 2009 (May)	118	Gastroenteritis diagnosed as salmonellosis between 2007 and 2009	ND	[75]

## 5. Antibiotic resistance

The misuse of antibiotics in humans and animals has for instance led to further amplification of antibiotic resistance thus leading to the global emergence of MDR fluoroquinolone-resistant *Salmonella* in different food commodities [2],[37]. This emerging drug resistance most notably for crucial antibiotics like β-lactams is particularly a challenge in addressing bacterial infection in human beings [49]. The discovery and the use of extended-spectrum oxyimino cephalosporins in the 1980s resulted in the emergence of extended-spectrum β-lactamases (ESBLs); prominent types are the TEM, SHV, and CTX-M groups. The increasing trend of CTX-M-producing bacteria over the last decade poses a great challenge since these enzymes are involved in resistance to other classes of drugs as well [76]–[78].

*Salmonella* isolates in Oman had moderate to high co-resistance to antibiotics in various sources, which poses significant AMR concerns [44]. The studies from Qatar found multidrug resistance in *Salmonella* obtained from chicken and retail products [14],[45]. Saudi Arabia identified repeated cycles of *Salmonella* incidents, thus stressing the dilemma of AMR in food security [10],[35]. Studies conducted in Saudi Arabia identified resistant strains in ready-to-eat foods and chicken carcasses [36],[46],[47]. Research conducted in the UAE found antibiotic resistance in *Salmonella* isolates obtained from chicken meat, vegetables, and water [48]–[51],[54]. Kuwaitian research demonstrated that resistance to ciprofloxacin in NTS in human infection has grown over time [62]. In Dubai, Infections due to typhoid have emerged with resistance to some of the most effective antibiotics [75].

These regional data suggest that there is a dire need for good strategies in order to deal with AMR. Strengthening antibiotic policies, improving food safety and the purposeful use of antibiotics are some of the right measures that should be taken to adequately address this emerging threat. Multi-sectorial approach involving countries and stake holders is important to reduce risks associated with MDR *Salmonella* and protect the health of people.

## 6. Food safety situation in GCC countries

To determine the changes in the food safety situation regarding the occurrences of *Salmonella* in GCC countries as an improvement or deterioration, some attributes have to be taken into account. include surveillance data, breakdown in frequency of outbreaks, public health infrastructure enhancement policies, and regulation and legislation updates [79]. There has been an improvement in the capability of surveillance and reporting mechanisms in the many GCC countries. Perhaps the enhanced means of collection and distribution of information by the national and regional authorities in health have enhanced the identification and declaration of *Salmonella* cases [28]. Many adverts concern health, especially food hygiene and preparation, personal conduct when dealing with foods, and signs and symptoms. These activities belong to an attempt to increase public awareness and decrease the food poisoning rate including *Salmonella* [9]. Improvements in the microbiological methods for detection and reporting of the source of contamination and a focus on various investigations into epidemiological factors have assisted in identifying and documenting cases of *Salmonella*. Also, centralized and regional laboratories are better placed to affirm infections and analyze infection trends [28]. Some policies that have received attention to enhance food safety regulation, are often in the form of measures that enhance safety features such as the processing, preparing, and handling of foods.

The fact that there have been increased reported incidences in certain areas may not be a result of the disease's growth but because of improvements in diagnosis and reporting. This has to do with enhanced laboratory capacity and surveillance systems to have accurate data [30],[49]. *Salmonella* is not evenly distributed in the GCC region, there are differences between the member nations. Some areas might register a higher number of cases due to variances in the approach to food preparation, the rate of development of the local public health systems, and efficiency in monitoring the disease [5]. There is evidence that public health campaigns and capacity-building efforts have brought some successes in some aspects to minimize the risk of transmission and better health outcomes [5],[7],[9],[35].

Overall, the status of food safety for *Salmonella* in the context of the GCC is revealing an upward trend that is owed to improvement in the country's surveillance networks, public health infrastructures, and muscular policy initiatives [30]. However, several areas need enhancement, including the need for continuous public education, more attention needs to be provided to food hygiene and safety, and the health systems in the countries need to be strengthened [7],[35]. Despite the symptoms of enhancement regarding the food safety situation of *Salmonella* in the GCC countries, the continued endeavors are vital to bring further advanced changes. Thus, it is crucial to enhance surveillance measures, educate the populace on the impacts of the bacteria, and enforce policies in a bid to prevent the occurrence of *Salmonella* in foods.

## 7. Discussion

*Salmonella* is a bacterium in the Enterobacteriaceae family and is classified as a gram-negative rod-shaped bacterium. It mainly affects humans through the use of foodstuffs of animal origin such as beef, chicken meat, pork, eggs, and milk products [7]. GCC countries are involved in systematic *Salmonella* surveillance at various levels. National public health organizations such as ministries of health, are responsible for surveillance and obtaining the reported cases of each healthcare facility across the nation via data sharing [5]. Regional and local health departments also support case surveillance in their part of the country. Centralized laboratories partner with regional referrals and regional laboratories to detect *Salmonella* infections by microbiological tests, while, at the same time, epidemiologists investigate confirmed cases to establish the sources of the infections and shared characteristics [80]. Stakeholders cooperation is effective in managing the response and sharing data. Consumer education involves being sensitive to food hygiene and knowing the signs of a potentially foodborne illness. Interventions improving surveillance, setting up or strengthening healthcare facilities, and developing staff competencies, prevent *Salmonella*, thus limiting its transmission [81].

These studies establish a host of causes of *Salmonella* including impure water, fish, and poultry imported from various countries [46],[54]. Further, Kuwait highlighted issues related to AMR and practices of food handlers, while Oman focused on the necessity of surveillance given the heterogeneity of contamination sources [65],[66],[82]. The global trade poses more challenges to Qatar by having increased risks, therefore, calling for improved food safety measures, though Saudi Arabia continues to experience regular outbreaks, hence the need to exercise vigilance regarding multidrug-resistant strains [9]. However, in UAE, the detection of *Salmonella* in chicken meats has signaled the need for continued monitoring; parks that use treated sewage water are also a cause for concern, and more efforts should be made to create awareness on hygiene [49],[54]. There is a dire need for increased vigilance in preventing *Salmonella*-related outbreaks and protecting public health, especially in areas such as Bahrain and Kuwait; restaurants should be urged to adhere to strict measures of hygiene and food safety [5]. Bahraini, Kuwaiti, and Saudi data on the incidence of salmonellosis inform the interventions, whereas Kuwaiti and Saudi studies focus on antimicrobial resistance and recommend stewardship programs. Kuwait and Qatari studies also stress food handler training as an effective intervention against contamination, while Oman and Qatari studies reveal aspects of the environment like sewage contamination [69],[82]. This has underlined the need for multiagency cooperation for the effective prevention of food-borne illnesses in Qatar and the UAE through increased awareness and policy enforcement on food hygiene [7]. Unfortunately, more specific information concerning hospitalization, failure of antibiotic therapy, and mortality due to *Salmonella* infections in GCC countries is lacking [10]. Comprehensive case reviews assessing the actual influence of *Salmonella* on hospitalization and mortality in the area are limited. Hence, there is a significant lack of information regarding the incidence of *Salmonella*-associated diseases, especially the hospitalization and mortality ratios in the GCC countries [10],[83].

Improvement in monitoring and surveillance of *Salmonella* contamination in the food chain from production to consumption is highly recommended. This entails having sound food safety policies and procedures to detect risk factors and prevent contamination incidents, including contaminated water and imported foods. Specific measures have to be taken to reduce set and particular risk factors leading to *Salmonella* outbreaks, the issue of antibiotic resistance, cyclical pattern of contamination rates, and food handler practices [10].

The approaches should thus include the implementation of overall antibiotic stewardship programs and check-ups of the antibiotic resistance profile. Teaching aids for food handlers avert cross-transmission and can be served in the food industry in a tailored course. Other awareness-creation activities include public outcries aimed at ensuring that consumers change their eating habits and administrations in restaurants to improve hygiene. Furthermore, educating people regarding adverse effects related to environmental reservoirs of *Salmonella*, for instance, parks sprinkled with water containing treated sewage, is crucial [49].

In response to issues that arise concerning outbreaks the best and future knowledge, development, and research can be proposed. First, enhanced and standardized health surveillance systems in GCC countries the incidence rate and trends can better be compared, which would help in formulating more suitable public health strategies in the region. Second, more studies in the future should be directed towards the exploration of additional risk areas for contamination or high risk of *Salmonella* outbreaks in restaurants, hospitals, and food production units. Discrete studies pointing to food handling practices, sanitation standards, and the microbiological quality of foods, especially specialist foods supply chain products incriminated in infections are examined.

## 8. Conclusions

*Salmonella* infection is a current important public health problem in the GCC countries, with diverse risk factors and sources of contamination causing its spread. Improving reported rates of *Salmonella* increasing vigilance, adopting rigorous food safety standards, and combating antimicrobial resistance remain vital steps toward controlling the effects of salmonellosis and protecting the health of populations in the region. These findings clarify that understanding and investigating the issue of salmonellosis in the Gulf is not straightforward, owing to the multitude of factors that have to do with cross-contamination of foods, antimicrobial resistance, diffusion of bacteria in the environment, and culture. Bridging these gaps needs efforts of reinforcing surveillance, implementing specific control measures, extending legislation, and enacting efficient regulations to reduce the impact of *Salmonella* and protect the health of the populace.

## Use of AI tools declaration

The authors declare they have not used Artificial Intelligence (AI) tools in the creation of this article.
